# Transcriptomic analysis of choroidal neovascularization reveals dysregulation of immune and fibrosis pathways that are attenuated by a novel anti-fibrotic treatment

**DOI:** 10.1038/s41598-022-04845-4

**Published:** 2022-01-17

**Authors:** Alice Brandli, Fay L. Khong, Roy C. K. Kong, Darren J. Kelly, Erica L. Fletcher

**Affiliations:** 1 Department of Anatomy and Physiology, The University of Melbourne, Grattan St, Parkville, VIC 3010 Australia; 2 Department of Medicine, The University of Melbourne, St Vincent’s Hospital, Fitzroy, VIC 3065 Australia; 3 Occurx Pty Ltd, 31 Queen St, Melbourne, VIC 3000 Australia

**Keywords:** Macular degeneration, Gene expression

## Abstract

Neovascular AMD (nAMD) leads to vision loss and is a leading cause of visual impairment in the industrialised world. Current treatments that target blood vessel growth have not been able to treat subretinal fibrosis and nAMD patients continue to lose vision. The molecular mechanisms involved in the development of fibrotic lesions in nAMD are not well understood. The aim of this study was to further understand subretinal fibrosis in the laser photocoagulation model of choroidal neovascularization (CNV) by studying the whole transcriptome of the RPE/choroid following CNV and the application of an anti-fibrotic following CNV. Seven days after laser induced CNV, RPE and choroid tissue was separated and underwent RNAseq. Differential expression analysis and pathway analysis revealed an over representation of immune signalling and fibrotic associated pathways in CNV compared to control RPE/choroid tissue. Comparisons between the mouse CNV model to human CNV revealed an overlap in upregulated expression for immune genes (Ccl2, Ccl8 and Cxcl9) and extracellular matrix remodeling genes (Comp, Lrcc15, Fndc1 and Thbs2). Comparisons between the CNV model and other fibrosis models showed an overlap of over 60% of genes upregulated in either lung or kidney mouse models of fibrosis. Treatment of CNV using a novel cinnamoyl anthranilate anti-fibrotic (OCX063) in the laser induced CNV model was selected as this class of drugs have previously been shown to target fibrosis. CNV lesion leakage and fibrosis was found to be reduced using OCX063 and gene expression of genes within the TGF-beta signalling pathway. Our findings show the presence of fibrosis gene expression pathways present in the laser induced CNV mouse model and that anti-fibrotic treatments offer the potential to reduce subretinal fibrosis in AMD.

## Introduction

Age-related macular degeneration (AMD) is the leading cause of irreversible vision loss in people aged over 55, in the industrialised world^1^. There are two forms of advanced AMD—an atrophic form called geographic atrophy that is characterized by death of photoreceptors and their support cells, the retinal pigment epithelium. The second form of advanced AMD is a neovascular type (nAMD), often referred to as “wet AMD”, that is characterized by pathological growth of blood vessels at the vision sensitive part of the retina, the macula. Untreated, nAMD can lead to severe vision loss, subretinal fibrosis and scarring. The introduction of treatments that target vascular endothelial growth factor (anti-VEGF) has substantially reduced vision loss in those with nAMD^2,3^. However, approximately 10% of patients do not respond to anti-VEGF, and in those treated long term approximately 50% lose significant levels of vision within 5 years, largely because of atrophy and/or the development of subretinal fibrosis^4–6^. To improve vision outcomes in those with nAMD, agents that target molecular signalling pathways involved in the development of these sequelae are required.

Fibrosis is a pathological state characterized by excessive deposition of extracellular matrix proteins such as collagen, fibronectin and laminin that are released by fibroblasts or myofibroblasts in response to injury^7^. From a clinical perspective, the presence of subretinal hyperreflective material (SHRM) is considered an important predictor of vision loss in nAMD^8–10^. In addition, there is pathological, and transcriptomic evidence suggesting that wound healing responses such as fibrosis develop alongside choroidal neovascularization^11^. Indeed, lesions associated with nAMD consist of both a neovascular component as well as an extravascular component consisting of fibrotic tissue, RPE cells, fibroblasts, myofibroblasts and inflammatory cells^12,13^. Transcriptomic studies on RNA isolated from humans eyes of those with nAMD have confirmed the upregulation of a range of genes associated with the extracellular matrix deposition^14,15^.

The molecular mechanisms involved in the development of fibrotic lesions in nAMD are not well understood. Fibroblasts are known to release signalling molecules that promote scarring, including platelet derived growth factor (PDGF) and tumour growth factor beta (TGFβ), both of which have been associated with CNV^16–18^. PDGF is known to promote fibrovascular lesions through the formation of myofibroblast-scaffolds, and the proliferation of RPE cells in a mouse model of CNV^16^. Inhibiting PDGF, via genetic knock down or drug antagonism reduces CNV pathology in the laser induced model of CNV^17,18^. In addition, clinical trials show that combination treatments targeting both PDGF and VEGF lead to greater improvement in visual outcomes than with anti-VEGF alone^19^. TGFβ, a well-known regulator of fibrosis, is expressed by a number of cell types in the posterior eye. Excised neovascular membranes isolated during submacular surgery have demonstrated that RPE cells express TGFβ^20,21^ as well as markers of trans-differentiation (i.e. Snail)^22^. In addition, circulating monocytes are reported to express higher levels of TGFβ in humans with nAMD^23^. Inhibitors of TGBβ signalling (e.g. decorin and tranilast) reduce pathology in the laser photocoagulation model of rodent CNV^24–26^ and recently reduction of TGFβ in microglia has been associated with reduced lesion size in the laser induced mouse model of CNV^27^.

Few studies have evaluated potential treatments of fibrosis in nAMD. Cinnamoyl anthranilates such as tranilast are known to have a broad range of effects that may include effects on TGFβ signalling and extracellular matrix turnover^28^. Notably, tranilast reduces proliferation of fibroblasts in vitro, and suppresses collagen deposition in vivo^29^ and has been used to reduce fibrosis in dermatological conditions such as scleroderma and keloid scarring (raised scar tissue)^30^. In addition to its anti-fibrotic properties, tranilast may also have anti-angiogenic properties owing to its role in inhibiting endothelial cell migration and tube formation. Treatment of rats with tranilast has been shown to reduce laser induced choroidal neovascularization^26^, suggesting that it may have beneficial effects in nAMD. We have synthesised a series of cinnamoyl anthranilate derivatives of tranilast that have superior potency and reduced toxicity compared to tranilast^31^. One of these derivates, FT011 (Fibrotech Therapeutics, Melbourne Australia) improves renal, cardiac and retinal function in a rat model of diabetes^32^. What is not known, however, is whether cinnamoyl anthranilates have potential to reduce fibrosis secondary to nAMD, and the mechanisms by which this might occur.

The aim of this study was to identify the dominant molecular signalling mechanisms associated with wound healing in the laser induced model of CNV and secondly, to determine whether cinnamoyl anthranilate reduces wound healing pathways identified in aim one and the development of fibrosis in the laser induced model of CNV. We performed RNA sequencing on the retinal pigment epithelium 7 days following laser induced CNV and undertook pathway and gene ontology analysis to determine the pathways activated during laser induced CNV. We then performed a series of experiments examining whether treatment at the time of laser induced pathway or 3 days later, reduced collagen and lesion size in the laser induced model. Overall, our results indicate an upregulation of immune and extracellular matrix remodelling pathways in CNV and overlap with other fibrotic models indicating the presence of fibrosis in laser induced CNV. In addition, treatment using a novel cinnamoyl anthranilate (OCX063) reduced CNV fibrosis pathology.

## Methods

### Animals

The mice used for all experiments were the C57Bl6J^ARC^ strain. Mice aged 6 weeks were obtained from the Animal Resource Centre, Perth, Western Australia and housed in the Biomedical Animal Facility at the University of Melbourne in cyclic light environment (12 h on, 12 h off, at an in-cage luminance of < 350 lux) for 10 or more days before use. 8-week-old mice were used for all experiments. All experimental methods and animal care procedures were approved by the University of Melbourne Animal Ethics Committee (#1513696) and in accordance with the ARRIVE guidelines. The study was carried out following all the relevant guidelines and regulations.

### Laser photocoagulation

Mice were anaesthetized with a combination of ketamine 67 mg/kg and xylazine 13 mg/kg via intraperitoneal injection. Corneas were anaesthetized with topical proparacaine hydrochloride (Alcaine; 0.5%, Alcon, Macquarie Park, NSW, Australia) and dilated with topical atropine 1% (Alcon). Corneas were lubricated with viscotears gel (Novartis, Macquarie Park, NSW, Australia) to prevent corneal drying. Four laser induced lesions were placed around the optic nerve (at the 3, 6, 9 and 12 o’clock positions) using an image guide laser photocoagulation system (Micron III, Phoenix Research Laboratories, Pleasanton, CA, USA; 532 nm continuous wave laser, 350 mW, 70 ms duration) as previously described^33^. Animals were intravitreally injected with a 0.5 μL aliquot of 400 μM cinnamoyl anthranilate derivative (final concentration 50 μM) (OCX063; OccuRx Pty Ltd, Melbourne, VIC, Australia) or sterile PBS using a 30-gauge needle either immediately following laser (day 7 endpoint) and 7 days following laser photocoagulation (day 30 endpoint).

### Fluorescein angiography and tissue collection

Seven days and 30 days following laser induced CNV, fluorescein angiography was performed to quantify the number and size of laser-induced lesions. Animals were anesthetized (ketamine 67 mg/kg and xylazine 13 mg/kg) and corneas were anaesthetized with topical proparacaine hydrochloride (Alcaine; 0.5%, Alcon) and dilated with topical atropine 1% (Alcon). Corneas were lubricated with viscotears gel (Novartis, Macquarie Park, NSW, Australia) to prevent corneal drying. Animals were injected subcutaneously with 100 μL of 1% sodium fluorescein (Fluorescite, Alcon). Brightfield and fluorescent fundus images were taken with the Micron III fundus camera 5 min following injection. Following fluorescein angiography, and whilst animals remained under anaesthetic, they were killed by cervical dislocation, their eyes removed and either placed in a fixative containing 60% Ethanol, 5% acetic acid, 4% paraformaldehyde, 3% sucrose in distilled water for histological analysis, or dissected and the RPE/choroid placed in a buffer containing RLT lysis buffer (Qiagen, Valencia, CA, USA) and 1% 2-mercaptoethanol and frozen at − 80 °C until required.

### Quantification of laser induced lesion size

Fluorescein angiogram fundus images were used to manually calculate the size of lesions in image J. For each eye, the four lesions were measured individually. Images were coded to allow for blinded measurements. In image J, selected images were processed by splitting into RGB channels. The green channel image underwent a background correction; a random unlasered area (200 pixel area) was selected between blood vessels and the threshold set to zero. Each lesion was quantified manually using the polygon tool to yield pixel area/lesion. Haemorrhage lesions and coalesced lesions were excluded from analysis based on either brightfield fundus images or pixel area/lesion > 20,000 pixels. Individual pixel/lesion measurements for each eye and treatment group were averaged and underwent statistical testing (one-way ANOVA).

### Immunohistochemistry for detection of collagen and trichrome staining

Following fixation, fixed eyes were dehydrated in an ethanol series, cleared with xylene, and embedded in paraffin wax using a tissue processor (Tissue-Tek VIP 5, Sakura Seikik Co., Ltd, Tokyo, Japan). Blocks were sectioned into 5 μm slices using a microtome. Sections labelled for collagen were dewaxed by immersion in Xylene, then processed through graded ethanol. Sections were incubated at room-temp for 24 h with anti-rabbit Collagen-IV (1:100, AB756P, Sigma, Castle Hill, NSW, Australia) in antibody diluent (1% bovine serum album, 0.05% triton in PBS). Sections were washed with PBS. Secondary antibody (1:500, goat anti-rabbit IgG alexafluor 594, Thermofisher) and DAPI (1:10,000, Sigma) was applied and incubated for 2 h at room temperature. Slides were rinsed in PBS and mounted in DAKO mounting media (Agilent, Mulgrave, VIC, Australia) and allowed to dry. Fluorescence images were captured using a Zeiss LSM700 confocal microscope (Zeiss, North Ryde, NSW, Australia) with no brightness or colour correction.

In order to quantify the extent of fibrosis within laser induced lesions, paraffin sections were prepared for staining with Masson’s trichrome stain. Briefly, sections were dewaxed by immersion in Xylene, then processed through graded ethanols, rinsed and then incubated in Bouin’s solution (70% Aqueous picric acid, 10% formalin and 5% acetic acid) at 60 °C for 1 h. Following further rinsing, slides were incubated in a 1:1 mix of Weigert’s iron haematoxylin solution (A: 1% w/v hematoxylin in 95% ethanol) and (B: 1.15% ferric chloride v/v, 1% hydrochloric acid in distilled water) for 2 min. Slides were then rinsed in water and stained with a 2:1 mix of Ponceau buffer (1% Ponceau 2R, 1% acetic acid in distilled water) and acid fuchsin buffer (1% acid fuchsin, 1% acetic acid in distilled water) for 5 min. Slides were then washed in water and immersed in phosphomolybdic acid (1% w/v phosphomolybdic acid in distilled water) for 3 min, followed by immersion in light green buffer (1% w/v light green SF yellowish, 1% acetic acid in distilled water) for 5 min, and lastly left in 1% acetic acid for 1 min. Slides were dehydrated in ethanol. Sections were cleared in fresh Xylene and mounted with D.P.X. and cover-slipped. Sections were imaged using a bright field microscope (Axioplan: Zeiss, Gottingen, Germany) using a 10 × objective. Digital images of each lesion were captured and quantified in Image J. Images were coded to allow for blinded measurements. Lesions were quantified by measuring the height of the lesion (b) and normalised to the choroid in an adjacent lesion-free area (c). The ratio of the lesion height to thickness of the choroid (b/c) was used to quantitatively assess changes in fibrosis across each treatment group.

### Bulk RNAseq and differential expression analysis

Total RNA was isolated from the RPE-choroidal samples using commercial spin columns (RNeasy, Qiagen). RNA purity was determined by UV absorbance using a Nanodrop spectrophotometer (ND-1000, Thermoscientific). RNA quantity and quality (RIN: RNA Integrity Number) was assessed with Tapestation (Tapestation 2200, Agilent). RNA yields were between 15–50 ng/µL totalling 150 to 500 ng of RNA per sample (n = 6 per group). Illumina's TruSeq total RNA sample preparation kit was used to prepare 23 libraries for sequencing in addition to ribosomal RNA (rRNA) depletion (Ribo-Zero Gold rRNA Removal Kit, illumine) both of which were performed by the Australian Genome Research Facility (Melbourne, Australia). Multiplex sequencing was performed on the Illumina HiSeq 2000 platform to obtain 50 bp single-end reads (averaging 17.3 million reads per library).

All sequenced data was assessed for quality using FASTQ software, and screened for the presence of any contaminants (adaptor sequences and cross-species sequences) and trimmed. Cleaned sequence data remaining was aligned to mouse genome (build version mm10) using TopHat software (v2.0.14) against gene annotations obtained from the Ensembl (v81 database). Stringtie (v1.2.4) was used for obtaining tag counts for each annotated gene using M6 and reference annotation based assembly option (RABT). Tag counts were normalised for library composition and library size using the default trimmed mean of m-values (TMM) normalisation method as implanted in EdgeR package (v3.18.1) to obtain counts per million (CPM). A generalised linear model was then used to quantify the differential expression comparison between the groups: control vs. CNV.

Using EdgeR (a generalised linear model), differential expression for the comparison (control samples vs. CNV samples) was calculated for each gene; the p-value, false discovery rate (FDR), log FC (fold change) and log CPM (counts per million). Genes lists for each comparison were generated by classifying genes that reached the following criteria (1) p < 0.05 and log2 ± 1.5 FC or (2) p < 0.05. Gene ontology and pathways associated with the gene list were determined by over-representation testing (PANTHER) for criteria 1 and 2. Gene ontologies were ranked based on FDR.

### Quantitative reverse transcriptase-PCR analysis

Total RNA was isolated from the RPE-choroidal samples using commercial spin columns (RNeasy, Qiagen). RNA purity and concentration were determined by UV absorbance using a Nanodrop spectrophotometer (ND-1000, Thermoscientific). An 84 gene PCR array (RT2-profiler, Qiagen) was used to assess the expression of angiogenesis and fibrosis genes. Total RNA samples from untreated-PBS injected, untreated-OCX063 injected, PBS-injected-laser-treated eyes and OCX063-injected-laser-treated eyes (n = 9 each group were pooled within their respective treatment groups to produce 3 sub-groups containing 75 ng RNA each). These samples (total 12 = 4 treatment groups × 3 sub-groups) were reverse transcribed to cDNA (RT2 first strand; Qiagen) and then underwent preamplification of the cDNA target templates (RT2 pre-AMP; Qiagen). Samples were added to a commercial master mix (RT2 SYBR green master mix; Qiagen) and amplified for 40 cycles (ViiA7: Thermoscientific). Three independent arrays were performed for each treatment group. Fold change was calculated using the ΔΔCt method, wherein the expression of the target gene was normalised to the relative expression of two reference genes—glyceraldehyde-3-phosphate dehydrogenase (Gapdh), and Peptidylprolyl isomerase A (Ppia).

### Statistical analysis

All results are expressed as the mean ± SEM. Statistical comparisons between the different treatment groups was performed using one-way ANOVA with appropriate post hoc analysis. Differences were considered statistically significant at p < 0.05. Analysis was performed in Graphpad prism version 9 (GraphPad Software, San Diego, California, USA).

## Results

**Figure 1:**
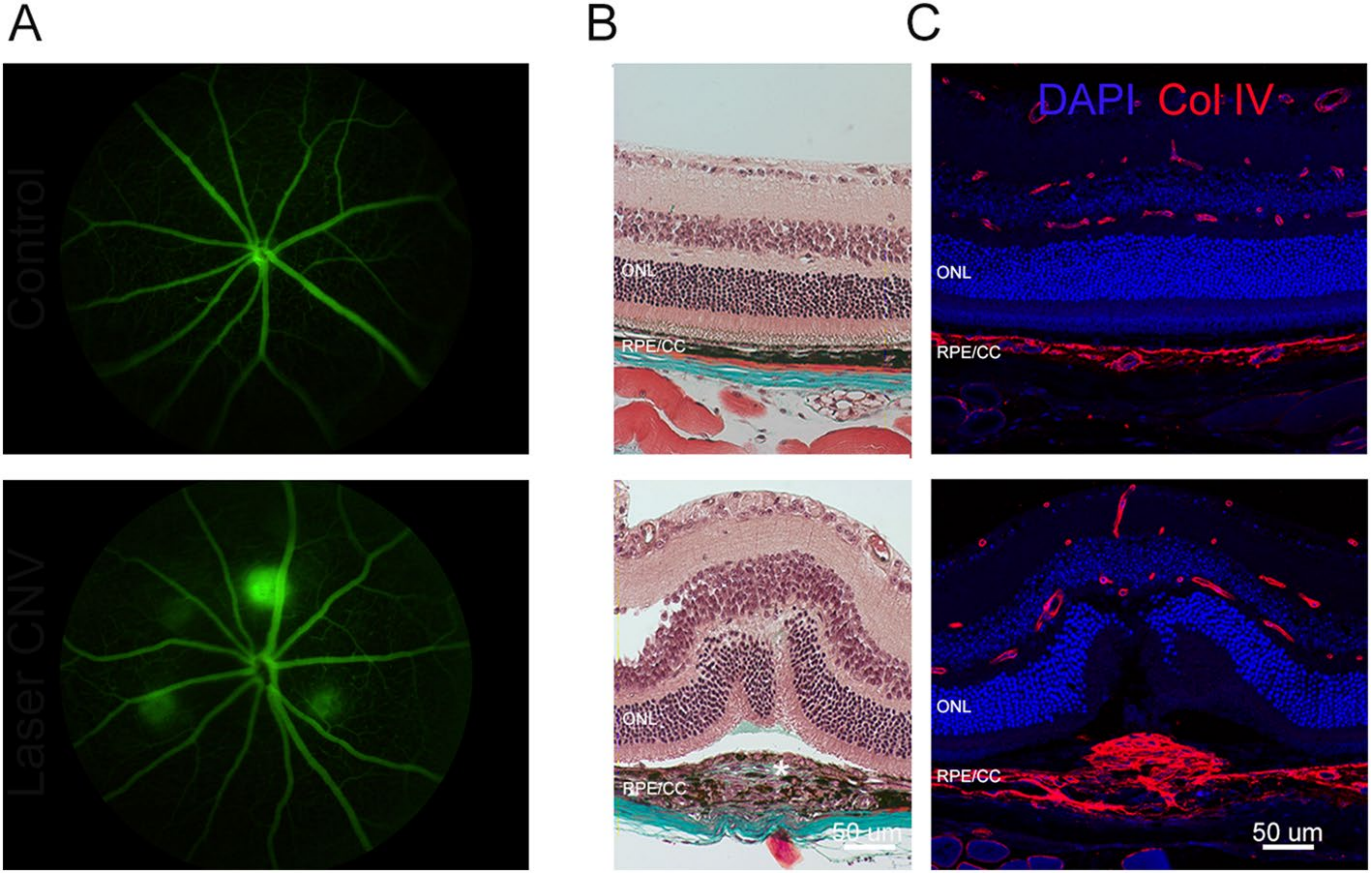
Fibrovascular characteristics of the laser CNV model in mice. (**A**) Fluorescein fundus images of a control eye upper panel and an eye 7 days after laser induced CNV in the lower panel. In the lower panel, there are four lesions visible around the optic nerve head. (**B**) Brightfield micrographs of vertical sections of retina stained with trichrome. The upper panel shows a control eye, collagen labelling (blue) is visible in sclera. Lower panel shows a CNV lesion with collagen visible (blue label) in sclera and within the lesion (white asterisk). (**C**) Fluorescent micrographs of vertical sections of retina stained with a nuclear stain (DAPI—blue) and collagen IV labelling (Col IV—red). The upper panel shows a control eye with collagen IV labelling within blood vessels of the retina and choroid. The lower panel shows collagen IV labelling in retina, choroid and a fibrotic mass of collagen within the CNV lesion. *CNV* choroidal neovascularization. Scale—50 μm.

In this study we used the laser induced model of CNV to study fibrosis and angiogenesis. As shown in Fig. 1A, four CNV lesions were induced and leakage analysis was performed 7 days later. We first confirmed the extent of fibrosis that develops in this model using Masson’s trichrome stain, where teal staining is visible within lesions indicative of an increase in collagen (Fig. 1B)^34^. As shown in Fig. 1C, collagen expression within lesions was confirmed by immunocytochemical labelling.

### RNAseq analysis and global gene expression of RPE–choroid complex following CNV

We next evaluated changes in the RNA expression of the RPE–choroid using RNAseq 7 days after laser induced CNV. RNAseq was performed on total RNA isolated from control (n = 6) and CNV groups (n = 5). The quality of RNA was > 8.0 RIN with a range of 150–500 ng of RNA per sample. Each library was derived from individual RPE–choroid RNA of different animals on the Illumina HiSeq 2000 platform to obtain 50 bp single-end reads (averaging 16.4 million reads per library, Table 1 in S1). The reads had high sequence coverage with 89% of reads aligned (range 89.1–90.4%) to the mouse (*M. musculus*) transcriptome.

**Figure 2:**
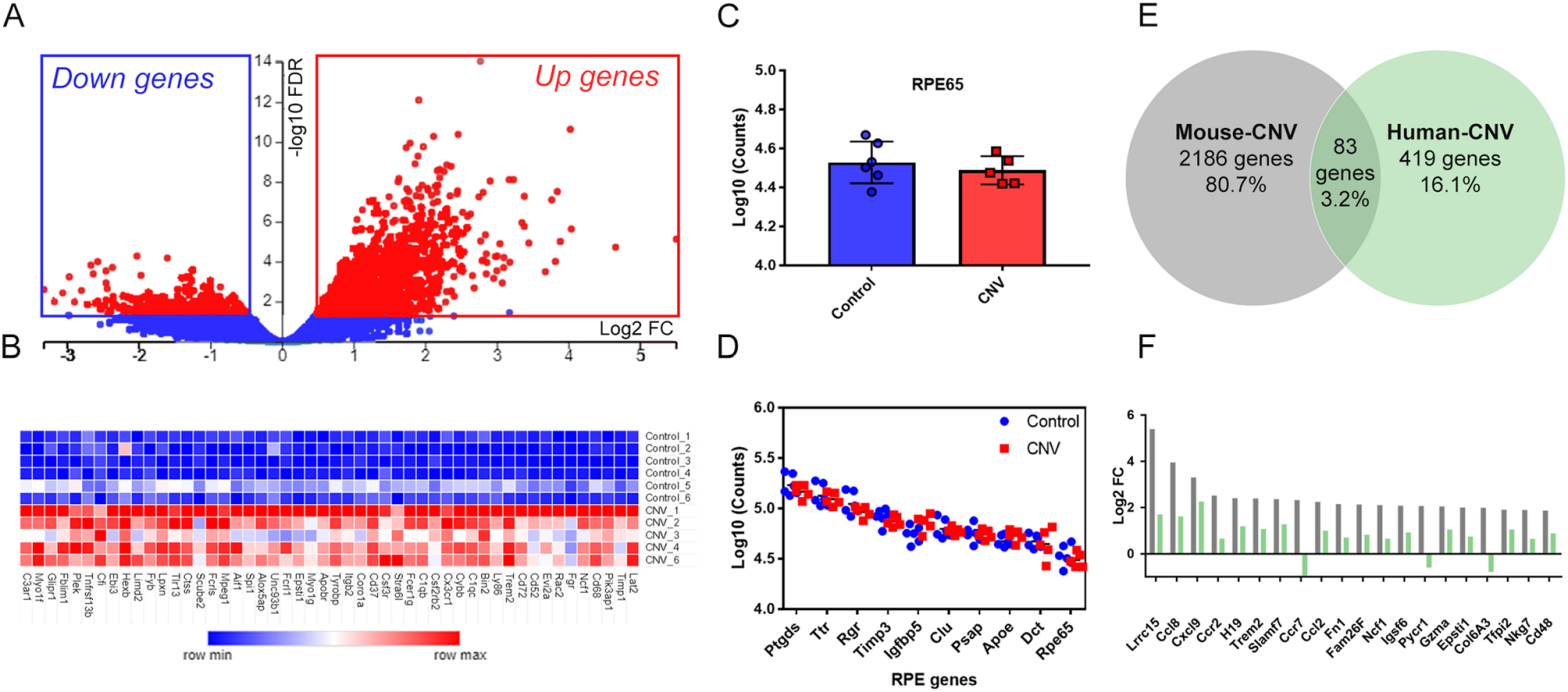
Whole genome expression of mouse RPE/Choroid tissue in CNV and similarities with Human-CNV. (**A**) A volcano plot shows that CNV resulted in upregulation of many differential expressed genes that were statistically significant: FDR < 0.05 (red) vs. non-significant: FDR > 0.05 (blue). x-axis is biological significance (log2 FC) and y-axis is statistical significance (− log10FDR). (**B**) A heatmap of the top 50 upregulated genes as ranked by biological significance. x-axis is the gene list ranked by fold change and y-axis is the list of samples grouped by control and CNV. The raw counts for each sample are shaded in pseudo colour with higher counts (red) and lower counts (blue). (**C**) Bar graph comparing RPE65 counts between control and CNV samples. x-axis is treatment groups and y-axis is log10 counts. (**D**) Log10 Counts for RPE signature genes indicating that the extraction method was enriched for RPE cells and equivalent between control (blue) and CNV (red) samples. x-axis is RPE signature genes ranked by count level and y-axis is log10 counts. (**E**) A Venn diagram showing the overlap of 83 genes between our mouse-CNV differential expressed genes (2186 genes, grey circle) and human-CNV (419 genes, green circle). (**F**) A bar graph showing top 10 genes ranked by biological significance that overlapped between mouse-CNV (grey) and human-CNV (green). x-axis genes ranked by mouse-CNV positive fold change and y-axis is log2 FC. *CNV* choroidal neovascularization, *FC* fold change, *FDR* false discovery rate. RNAseq performed on n = 5 CNV samples and n = 6 control samples.

Differences in gene expression between control and CNV was performed using EdgeR. For each gene; the log CPM p-value (counts per million), p-value, false discovery rate (FDR) and log FC (fold change) was calculated. The library size and normalised gene expression was similar across the 11 samples demonstrating equivalent distribution of counts across samples (Supplement 1 Table S1). A multidimensional scaling (MDS) plot of the EdgeR expression data indicates sample similarity based on distance between samples. In Supplement 1 Fig. S1, the biological replicates of the control and CNV groups were clustered into two distinct groups across the x-axis (MDS dimension 1). In order to account for multiple sampling of data, we applied a false discovery rate (FDR) of less than 0.05, and, using this criterion, identified 2186 significantly expressed genes. The volcano plot shown in Fig. 2A displays genes along dimensions of biological (y-axis) and statistical significance (x-axis) with significant genes (FDR < 0.05 in red) and non-significant genes (FDR > 0.05 in blue).

There were 2186 genes that were significantly dysregulated (FDR < 0.05). Most significant genes were upregulated (1791 genes) than downregulated (394 genes). The 50 most upregulated genes as ranked by significance are displayed in a heatmap (Fig. 2B) and the full list of significant genes are shown in Supplement 2 Table S2. Many of the upregulated genes reflect infiltration of monocyte macrophages (e.g. Cd68, Mpeg1, Aif1), microglia (e.g. Cx3cr1), neutrophils (e.g. Ncf1) and leukocytes (e.g. Glipr1, CD37) into the CNV lesion site as previously reported^35–38^. There was also upregulation of components of the complement pathways (C1qb, C1q, CFI, C3ar1) consistent with previous mice CNV gene expression studies^39,40^ and also chemokines (*Ccl2*,* Ccl9*), collagen (*Col1a1*, *Col3a1*, *Col4a1*) and genes associated with the extracellular matrix (timp1, Mmp3, Mmp12)^41^.

Genes known to be associated with RPE cells were highly abundant, for example, RPE65, a gene uniquely expressed in the RPE was found to be equally expressed in both control and CNV treated tissue (Fig. 2C). “RPE signature” genes of which 10 have been identified in gene expressional studies of cultured and ex-vivo human RPE as well as mouse RPE^42,43^.The highest gene counts were observed for RPE signature genes Ptgds, Trt, Rgr, Timp3, Igfbp5, Clu, Psap, Apoe, Dct, and RPE65 (range 50,000–150,000 counts per sample, the log_10_ values were plotted, Fig. 2D). Overall, for the 10 RPE signature genes counts were equally varied between control and CNV samples (control var = 1.36 × 10^9^ counts and CNV var = 2.54 × 10^9^ counts, Fig. 2D) indicating that the treatment and control samples were both enriched for markers of RPE cells.

We next evaluated whether the differentially regulated genes identified following laser induced CNV were similar to those altered in human nAMD tissue (RPE/Choroid). The gene data from Newman et al. was converted to log_2_fold change and FDR 0.05^14^. As shown in Fig. 2E, there were 83 differentially expressed genes (or 3% overlap) that were common to both human nAMD and mouse CNV^14^. The full gene list is found in Supplement 2 Table S3. Of the 83 genes, Lrcc15, Cxcl9, Ccl8, Cccl2, Comp, Fndc1, thbs2 were the highest ranked genes in both human and mouse (the 20 highest differentially regulated genes (by fold change) are plotted in Fig. 2F). Overall, the results suggest that there are a significant number of commonly altered genes in the laser induced mouse model of CNV and human nAMD.

### Pathway analysis of RPE–choroid complex gene expression following CNV

**Figure 3:**
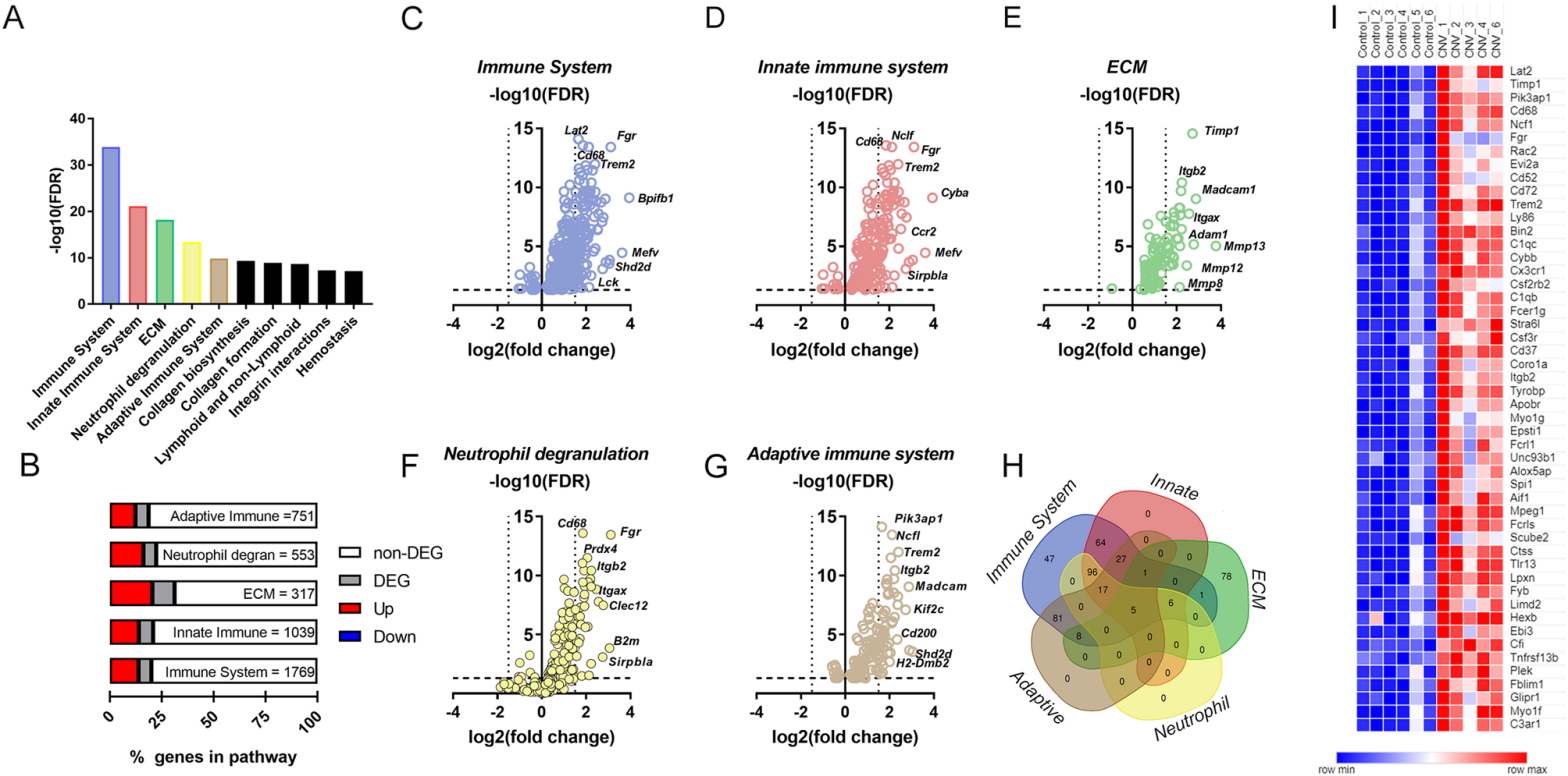
Immune system and extracellular matrix reactome pathways were identified in overrepresentation testing of DEG mouse-CNV genes. (**A**) Bar graph showing the top 10 reactome pathways ranked by statistical significance. x-axis is reactomes ranked by statistical significance (FDR) and y-axis is − log10 FDR. The top five reactomes are further analysed; immune system (blue), innate immune system (red), ECM (green), neutrophil degranulation (yellow), adaptive immune system (brown). (**B**) Bar distribution graph showing that ECM reactome has the highest percentage of DEG expressed compared to the other top five reactomes. x-axis is % of genes in pathway. Legend: non-DEG genes (white), DEG (grey), up regulated DEG (red) and down regulated DEG (blue). (**C**) Volcano plot of immune system reactome genes (blue) that are DEG. x-axis is biological significance (log2FC) and y-axis is statistical significance (− log10 FDR). (**D**) Volcano plot of innate immune system reactome genes (red) that are DEG. (**E**) Volcano plot of ECM genes (green) that are DEG. (**F**) Volcano plot of neutrophil reactome genes (yellow) that are DEG. (**G**) Volcano plot of adaptive immune system reactome genes (brown) that are DEG. (**H**) Venn diagram that overlaps immune system (blue), innate immune system (red), ECM (green), neutrophil degranulation (yellow), adaptive immune system (brown) reactomes to show there is little overlap between ECM and the immune-related pathways. (**I**) Heat map of the upregulated genes in the ECM reactome. x-axis is the samples grouped by control and CNV and y-axis is genes ranked alphabetically. The raw counts for each sample are shaded in pseudo colour with higher counts (red) and lower counts (blue). *DEG* differentially expressed genes, *ECM* extracellular matrix, *FDR* false discovery rate.

We assessed the functional implications of the significantly differentially expressed genes (2206 mapped genes) by identifying gene ontologies (GO) on the PANTHER platform for reactome pathways^44^. We took a non-biased approached to study gene ontologies with over-representation statistical testing revealing 105 significant reactome pathways (Supplement 2 Table S4). The top 10 pathways ranked by FDR are displayed in Fig. 3A. The highest ranked pathways include the immune system, innate immune system, extracellular matrix (ECM), neutrophil degranulation and the adaptive immune system. The significant genes for each pathway were graphed as volcano plots in Fig. 3C–G. Of these pathways, the ECM gene cluster contained the highest proportion of upregulated genes (20.5% upregulated genes; range for top 5 reactomes = 12.4–20.5%) (Fig. 3B). As noted in Fig. 3H, differentially expressed genes belonging to the ECM gene ontology had very little overlap with any of the four other differentially regulated pathways, highlighting that dysregulation of genes associated with the immune system and genes associated with the ECM represent two separate groups of genes that are upregulated in the laser induced model of CNV. There were also other extracellular remodelling pathways identified such as collagen biosynthesis and collagen formation. A heatmap of counts for significant ECM pathway genes (ID:R-HSA-1474244.2, 72 genes) is shown in Fig. 3I and demonstrates that in RPE/choroid extracted from laser CNV were higher than those extracted from control tissue.

**Figure 4:**
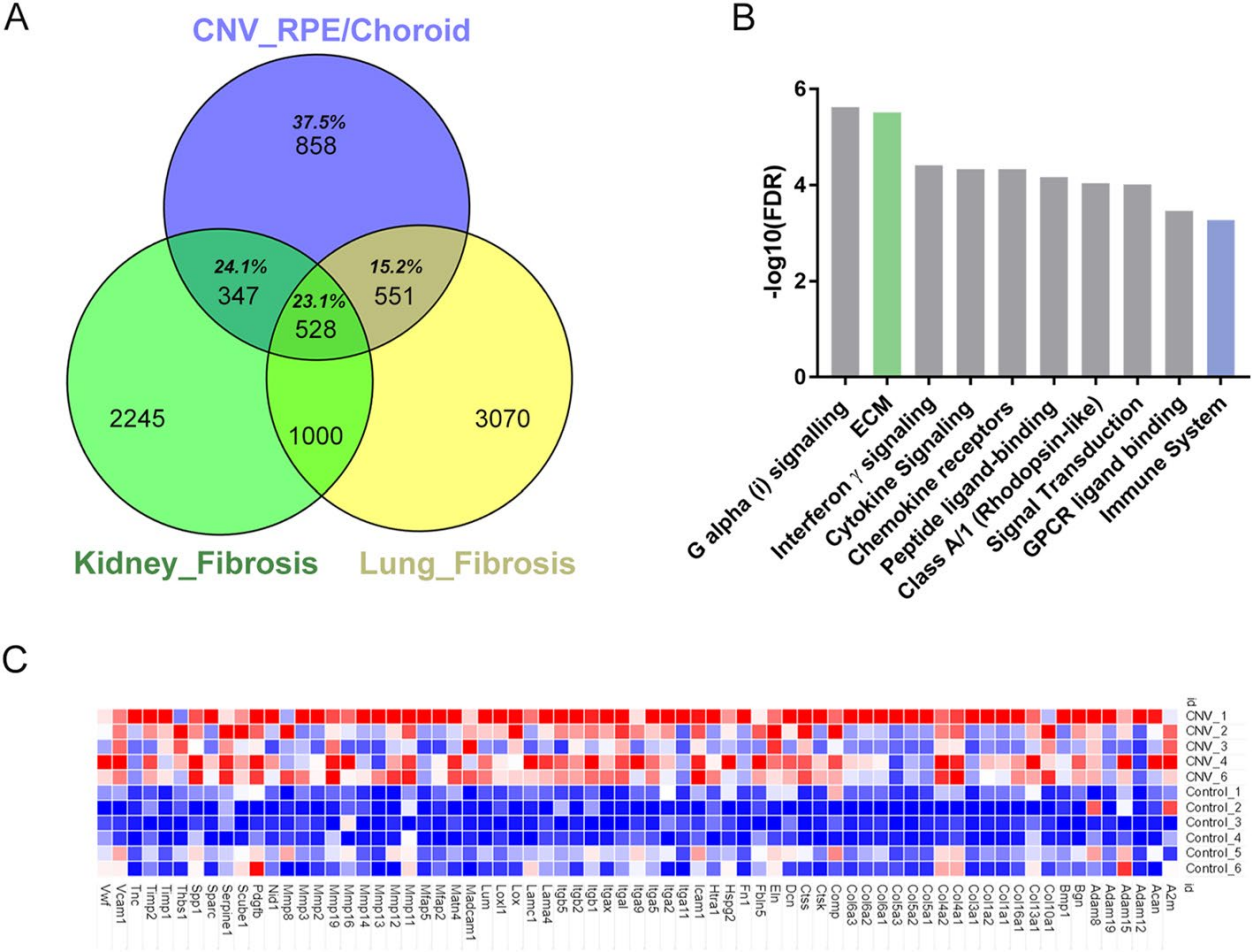
Overlap of DEG between CNV and other models of fibrosis and evidence of ECM gene signalling in human CNV. (**A**) Venn diagram showing that the majority of DEGs (total 62.5%) in laser CNV overlap (purple) with either kidney (teal), lung fibrosis (yellow) or both (green). (**B**) Reanalysis of human-CNV DEG genes shows that ECM pathway (green) was the second highest significant reactome compared to the other top 8 reactomes (grey) and immune system reactome (blue). x-axis is reactome pathways ranked by statistical significance (FDR) and y-axis is − log10 FDR. (**C**) A heatmap of RNAseq counts for the ECM reactome genes showing upregulation in CNV samples compared to control samples. The raw counts for each sample are shaded in pseudo colour with higher counts (red) and lower counts (blue). *DEG* differential expressed genes, *CNV* choroidal neovascularization, *FDR* false discovery rate.

Our observation that laser induced CNV was associated with changes in many genes associated with the ECM and collagen formations, implies that molecular pathways associated with fibrosis may be a feature of laser induced CNV. We next compared the differentially expressed genes identified in the laser induced CNV model with publicly available datasets of mouse models of fibrosis—including lung fibrosis and kidney fibrosis^45,46^. The model of lung fibrosis involved changes induced 7 days after injection of Bleomycin, a well-accepted model of interstitial pulmonary fibrosis. Kidney fibrosis was induced by surgical obstruction of the ureter and tissue collected 8 days after obstruction. The model of kidney fibrosis was associated with 5149 significantly dysregulated genes (p < 0.05, no FDRs calculated) from a RNAseq study and the lung model contained 4120 genes (adjusted p < 0.05) from a DNA microarray study. There was considerable overlap between the gene lists generated from all three tissues (Fig. 4A). The majority of CNV upregulated genes were common to fibrosis models with 1426 genes or 62.5% overlapping with either kidney fibrosis (347 genes, 24.1%), lung fibrosis (551 genes, 15.2%) or lung and kidney fibrosis (528 genes, 23.1%). The high commonality of differentially regulated genes observed in both lung and kidney fibrosis suggests that fibrosis is present in CNV. We reanalysed the human nAMD gene dataset (512 genes with 459 genes mapped, p < 0.05)^14^ by over representation testing using PANTHER and revealed 18 significant reactome pathways (Supplement 2 Table S5). The genes of the extracellular matrix organisation (ECM) pathway for human CNV compared to mouse CNV is shown in Fig. 4C. The extracellular matrix organisation (ECM) pathway was second highest pathway (Fig. 4B) and dysregulation in ECM indicates fibrosis^47^. The over-representation of the ECM pathway in human nAMD, mouse CNV and in animal models of fibrosis indicates that ECM fibrotic signalling in present nAMD.

### Treatment of CNV with cinnamoyl anthranilates known to target ECM remodelling and anti-immune signalling

RNA sequencing revealed that genes associated with immune function and extracellular matrix formation were differentially regulated in the laser induced model of CNV. Therapeutic agents, including tranilast and other cinnamoyl anthranilates, are known to reduce fibrosis by altering ECM remodelling^28^. Therefore, we evaluated the potential for the cinnamoyl anthranilate, OCX063 to reduce pathology in the laser induced mouse model of CNV. The potential of OCX063 to treat CNV was studied by intravitreally injecting OCX063 or vehicle (PBS) immediately following laser injury with retinal effects assessed at 7 days and 30 days later. The later time point was selected, because previous studies suggested that fibrosis increases over time^48^.

**Figure 5:**
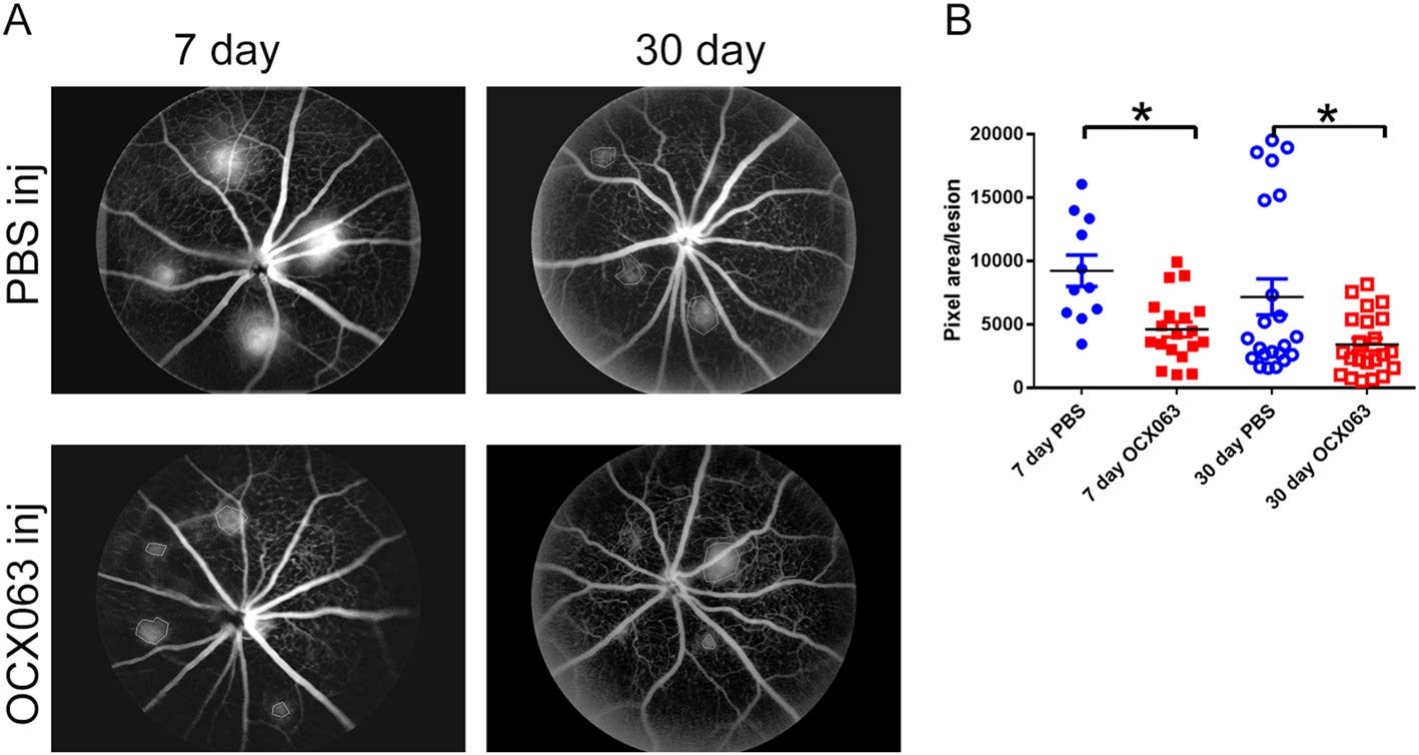
OCX063 reduced fluoresceine leakage 7-days and 30-days following laser induced CNV. (**A**) Grey scale fundus images that show leakage size is smaller in OCX063 injected mice compared to sham (PBS) injected eyes at 7- and 30-days post laser induced CNV. Upper panels are PBS injected eyes. Lower panels are OCX063 injected eyes. (**B**) Graph showing pixel area/lesion was statistically smaller in OCX063 injected mice (red squares filled: 7 days, red square outline: 30 days) compared to PBS injected eyes (blue circles filled: 7 days, blue circles outline: 30 days). x-axis is treatment groups and y-axis is pixel area/lesion. Error bars ± SEM, *p < 0.05 Tukey’s post-hoc following one-way ANOVA.

**Figure 6:**
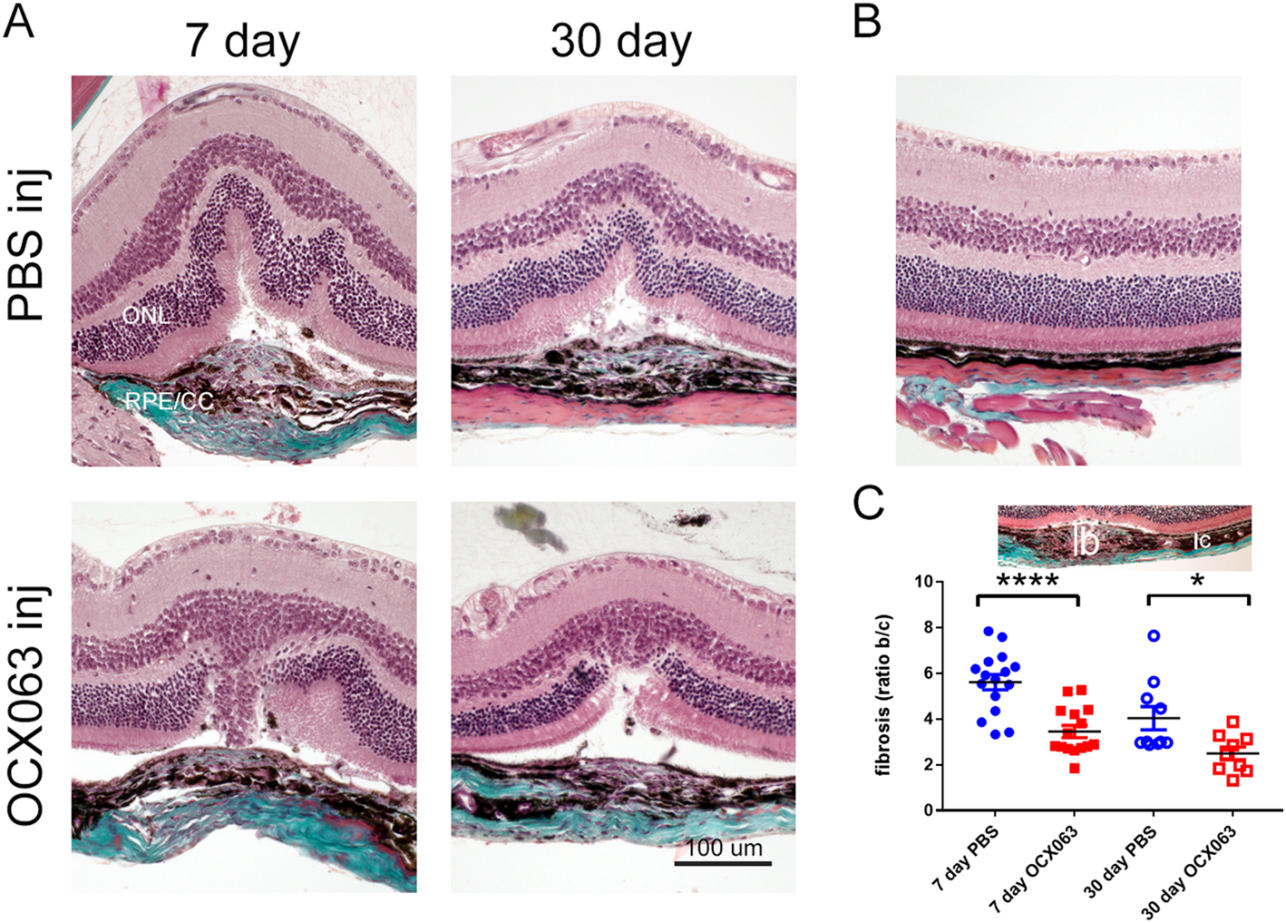
OCX063 reduced fibrovascular lesion size 7-days and 30-days following laser induced CNV. (**A**) Brightfield images of laser induced CNV retina with trichrome staining showing that fibrovascular lesions size was reduced with OCX063 treatment compared to PBS injected at both 7- and 30-days post CNV. Trichrome labels collagen (teal) and staining is visible in centre of fibrovascular lesion and in sclera were visible. Upper panel are PBS injected. Lower panel are OCX063 injected. (**B**) Brightfield image of control retina stained with trichrome showing trichrome staining limited to sclera. (**C**) Graph showing fibrosis (ratio B/C) was statistically smaller in OCX063 injected mice (red squares filled: 7 days, red square outline: 30 days) compared to PBS injected eyes (blue circles filled: 7 days, blue circles outline: 30 days). x-axis is treatment groups and y-axis is lesion ratio b/c. Insert shows lesion height measurements, *b* lesion and *c* choroid. Error bars ± SEM, *p < 0.05 and ****p < 0.0005 Tukey’s post-hoc following one-way ANOVA (n = 9 animals per group). *B* lesion height, *C* choroidal thickness, *ONL* outer nuclear layer, *RPE/CC* retinal pigment epithelium/choroid capillaries. Scale = 100 μm.

As shown in Fig. 5, treatment with OCX063 reduced lesion size as indicated by the reduced leakage at both 7- and 30-days following laser induced CNV. Lesions were smaller on average for 7-days (average pixels: PBS 9211 pixels ± 4076 S.D. vs. OCX063: 4601 pixels ± 2564, p = 0.041 Tukey’s post-hoc analysis) and 30-days (average pixels: PBS 7165 pixels ± 6686 S.D. vs. OCX063 3406 pixels ± 2369, p = 0.0281 Tukey’s post-hoc analysis). The presence of fibrotic scar tissue was detected using Masson’s trichrome staining which labels collagen fibres in blue. Trichrome labelling of normal mouse eyes shows the normal presence of collagen fibres in the sclera (Fig. 6B) and in lasered eyes abnormal deposits of collagen were observed at the centre of CNV lesions (Figs. 1 and 6A). We quantified fibrosis by measuring the lesion height of trichrome positive staining lesion relative (B) to the thickness of the adjacent normal choroid (C) for fibrosis (ratio B/C) for each of the treatment groups (Fig. 6C). Injection of OCX063 reduced fibrotic lesions at the 7-day time point (average B/C ratio; PBS: 5.6 ± 1.3 S.D. vs. OCX063: 3.45 ± 1.02 S.D., p < 0.0005 Tukey’s post-hoc analysis) and at the 30-day time point (average B/C ratio; PBS: 4.0 ± 1.6 S.D. vs. OCX063: 2.49 ± 0.8 S.D., p = 0.045 Tukey’s post-hoc analysis) relative to PBS injection (Fig. 6). In summary, intravitreal injection of the cinnamoyl anthranilate, OCX063, reduced laser induced lesion leakage and the fibrovascular lesion size compared to PBS injection.

In view of the reduction in laser induced pathology by OCX063, we anticipated that gene expressional changes altered in the RPE–choroid in response to laser induced CNV would be abrogated by treatment with OCX063. To test this, gene expressional studies were conducted using PCR arrays containing either 84 genes associated with fibrosis or 84 genes associated with angiogenesis. As shown in Supplement 2 Table S6, laser induced CNV was associated with a greater number of differentially expressed genes in the RPE than the retina. As shown in Supplement 1 Fig. S2, of the differentially expressed genes (p < 0.05, FC ± 1.5) two were immune related genes (Ccl3 and il13ra2), two were associated with angiogenesis; (Thbs1 and Anpep), and four were ECM genes (Col1a2, Mmp13, Mmp8, Timp1). Importantly, 10 genes differentially expressed in the RPE were similar in direction and magnitude as those identified in the RNAseq data (Supplement 1 Table S7).

**Figure 7:**
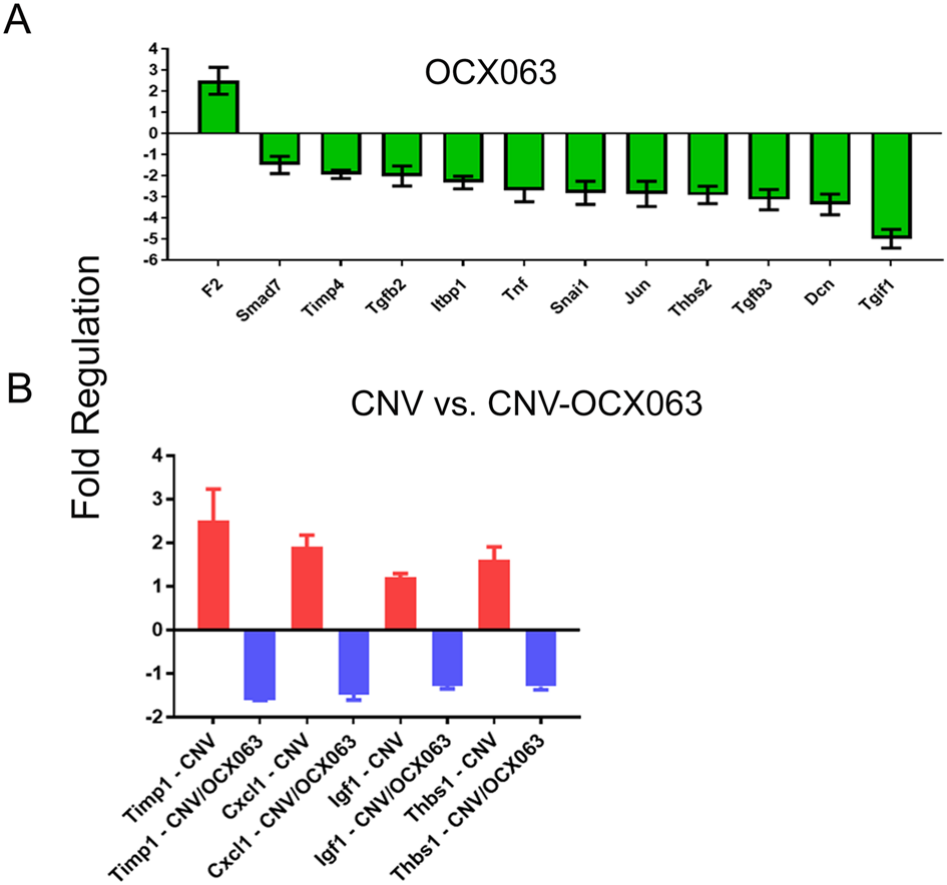
OCX063 reduced mRNA expression of TGFβ signalling genes in control and fibrovascular lesion size 7-days and 30-days following laser induced CNV. (**A**) Bar graph of OCX063 regulated gene in non-lasered RPE/choroid (green). (**B**) Bar graph of regulated genes in OCX063 regulated genes in lasered RPE/choroid (blue) compared to lasered RPE/choroid (red). x-axis genes ranked by fold change positive to negative and y-axis is fold change. n = 9 animals per group.

Treatment of laser induced CNV with OCX063 resulted in a change in gene expression in four genes in the RPE (Fig. 7B). OCX063 reduced expression of the ECM genes; Timp1 and Thbs1 the cytokine; Cxcl10 and insulin growth factor Ig1f. Based on these results, it is likely that OCX063 modifies the expression of ECM genes and has a limited effect on the immune environment of the RPE–choroid during CNV. In non-lasered mice, OCX063 also affected the ECM in the absence of fibrosis. Indeed, OCX063 reduced expression in non-lasered mice for Timp4, and genes from the TGFβ family including Smad7, Snai1, Tgfb2, Tgfb3 and Tnf (Fig. 7A). Several studies have demonstrated that TGFβ signalling participates in the development of CNV pathology in the laser induced model by activating VEGF signalling. In our RNAseq study of CNV, the TGFβ pathway was enriched (FDR = 0.01, Fold enrichment 2.32 with 22 genes expressed out of 103 genes within the pathway GO: 0007179) and in the qPCR study, Tgfb1 was overexpressed. Taken together, these data suggest that OCX063, as an anti-fibrotic agent, may act through the TGFβ pathway to reduce CNV pathology.

## Discussion

The major findings of this study were that a range of complex molecular signalling pathways associated with immune and fibrosis signalling were upregulated within 7 days of laser induced CNV. Notably, the molecular changes were similar to those identified in human CNV, as well as a number of other models of organ fibrosis, suggesting that there are common fibrotic pathways in kidney fibrosis, lung fibrosis and laser induced induced CNV^14,45,46^. Finally, treatment with the cinnamoyl anthranilate derivative of tranilast, OCX063, reduced laser induced pathology and attenuated gene expression of TGFβ associated genes. Overall, these results suggest that there are common molecular mechanisms that underpin fibrosis in several organs, and that using an anti-fibrotic treatment, such as OCX063, in laser CNV has potential to reduce fibrosis pathology.

Our study demonstrated the upregulation of immune-related pathways based by overrepresentation testing in laser CNV tissue. The immune related reactomes were immune system, innate immune system, adaptive immune system, neutrophil degranulation and lymphoid and non-lymphoid signalling. Upregulation of these pathways, and signature genes indicate that lymphocytes, macrophages and neutrophils that are not normally found in the RPE/choroid were present within CNV lesions. Macrophages and neutrophils have previously been shown to be present in the lesions of the RPE/Choroid^49^ and are understood to be essential in development of laser induced CNV^35,37^. The evidence that macrophages were increased in CNV RPE/choroid is based on the upregulation of macrophage genes including Ccr2, Ccl8, Cxcl9 and Cxcl10 that were also reported in a DNA microarray study of mouse CNV tissue^50^. Of note are the chemokines, Cxcl9/MIG and Cxcl10/IP-10 as they have been reported to be upregulated in in nAMD RPE/choroid tissues and elevated in aqueous humour or serum samples of patients with nAMD^14,51–54^.

The underlying mechanism(s) that drive fibrosis in nAMD are poorly understood. Lesions isolated from humans contain blood vessels, immune cells, fibroblast and myofibroblasts and an abundance of extracellular matrix (ECM) proteins such as collagen, fibronectin and laminin^55,56^. We find supporting evidence for fibrosis signalling in laser induced CNV tissue by using a RNAseq study. In this differential expression RNAseq study upregulation of ECM remodelling and collagen pathways in the CNV mice was observed. Under normal conditions the ECM provides a support structure between the RPE and choroid including Bruch’s membrane^57^. ECM regulation is an active process of building and disintegration of ECM components to maintain healthy tissue architecture. The components of ECM are proteoglycans (e.g. decorin and chondroitin sulphate), collagen fibres (e.g. Col I and Col IV) and extracellular proteins (e.g. fibronectin and laminin). The proteins involved in ECM remodelling are metalloproteinases, or matrix metalloproteinases (MMPs) and are regulated by tissue-inhibitor metalloproteinases (TIMPs) both of which are reported to be expressed at higher levels in excised nAMD lesions compared to age matched controls^58,59^. Mmp-2, Mmp-9, Mmp-13 have been shown to be upregulated in nAMD and the mouse model of CNV, further by inhibiting Mmp9 and Mmp-13 in mice models it was shown that CNV pathology reduces^59–61^. Inhibition of Timp-1 has also been shown to reduce neovascularization of mice over-expressing VEGF^62^. The ECM gene ontology was highly enriched with upregulation of Timp-1, MmpP-8 (neutrophil collagenase), MmpP-12 (macrophage metalloelastase) of which Mmp-13 (collagenase 3) had been previously reported in the context of CNV studies. The expression of these genes was stably expressed as later qPCR of RPE–choroid tissue also showed upregulation of Mmp-8, Mmp-13, Timp1 and the collagen gene Col1a. There were two ECM genes that were also reported by Newmann et al. to be upregulated in human, Thbs-2 (Thrombospondin-2) and Dcn (Decorin)^14^. Decorin is a proteoglycan that is found in the ECM of the RPE–choroid complex in humans^63^ and can also be found in endothelial cells that are undergoing angiogenesis^64^. Decorin is able to stabilise the ECM, suppress TGFβ by binding and depending on the context can either act as a pro-angiogenic or anti-angiogenic molecule^64,65^. Decorin has recently been targeted in the laser CNV model, where intravitreal delivery of decorin reduced CNV pathogenies and VEGF excretion^66^. The other key genes upregulated in out mouse CNV study were the collagen genes (Col1a2, Col1a1, Col3a1) that were consistent with a previous DNA microarray studies using the CNV mouse model^41^.

Our results demonstrate that a single application of OCX063, a cinnamoyl anthranilate derivative, at day 0 was able to reduce both vascular leakage and the size of the fibrovascular lesion at both day 7 and day 30. This indicates that OCX063 has anti-fibrotic and anti-inflammatory activity. The mechanism by which OCX063 reduces laser induced pathology was analysed. In the RNAseq analysis, it was shown that Tgfb1 and Tgif1 genes were upregulated in CNV consistent with another mouse CNV study that assessed protein expression of TGFβ^67^. The cells that release TGFβ in the context of choroidal neovascularization are; human RPE cells at nAMD lesions^21^, circulating monocytes in humans^23^, and endothelial cells in mouse CNV models^68^. Modifying TGFβ expression either through application of decorin (inhibitor) or synthetically inhibitors has consistently been reported to reduce CNV pathology^24,25,69^. TGFβ is considered a master regulator of fibrosis as it can alter ECM remodelling and fibroblast phenotype but also capable of promoting angiogenesis. The diversity in TGFβ affects can be understood to be due to the variation in Smad signalling via Alk5/Smad 2/3 and Smad 4 (fibrotic) or alternatively Alk1/Smad 1/5/8 (pro-angiogenic)^70^. Comparing OCX063 injection against PBS injected eyes revealed that Tgf-b1 gene signalling was suppressed across the pathway for TGFβ (Tgif1), Tgfb receptors 1–3 and the Smad receptor Snail and in the CNV-OCX063 group, down regulation of Tgif1 and Smad4. This provides strong evidence that OCX063 affects fibrotic signalling of TGFβ as seen in the reduction in the pro-fibrotic Smad4. The use of anti-fibrotics, specifically OCX063 was shown to reduce CNV pathology; for both vascular leakage and fibrotic scarring. Our gene expression studies showed OCX063 modified TGFβ signalling (a master regulator of fibrosis) and is a candidate for the development of novel treatments for nAMD.

In summary, suppression of TGFβ remains a promising target for the treatment of neovascular AMD due to it’s ability l to modulate fibrosis. In nAMD, fibrosis remains a difficult to treat pathology despite anti-VEGF therapies^5,6^. Although no animal model recapitulates all the features of nAMD, there are many similarities in the presentation of the CNV lesion in humans with respect to the presence of fibroblastic cells and pro-inflammatory cells. There was transcriptomic similarity between mouse CNV and human nAMD, notably in the chemokines used to signal orexpressed by macrophages and fibrosis markers such as Dcn and Lrrc15^71^. Further studies should be directed at targeting the ECM pathways activated in nAMD either TGFβ-dependent or independent to modify fibrosis and in turn prevent vision loss.

## Supplementary Information

Supplementary Information 1.

Supplementary Information 2.
