# Supplementary material for: Transcriptomic analysis of choroidal neovascularization reveals dysregulation of immune and fibrosis pathways that are attenuated by a novel anti-fibrotic treatment
Source: Sci Rep. 2022 Jan 17;12:859. doi: 10.1038/s41598-022-04845-4 (PMC8764037; doi:10.1038/s41598-022-04845-4)
Supplement: Supplementary file 1 — Supplementary Information 1. [file 41598_2022_4845_MOESM1_ESM.pdf]

Supplementary 1

Table S1: RNAseq library sizes of RPE/choroid tissue

Library	Samples in group	Read length (bp)	Total reads (million)	Number of reads mapped to genome	% genes mapped	% reads mapped to one feature
**Control**	6	50	17.9	16.7	89.5	68.9
**CNV**	5	50	14.8	14.1	89.8	70.5

Figure S1: Consistent RNAseq quality control across RPE/choroid samples

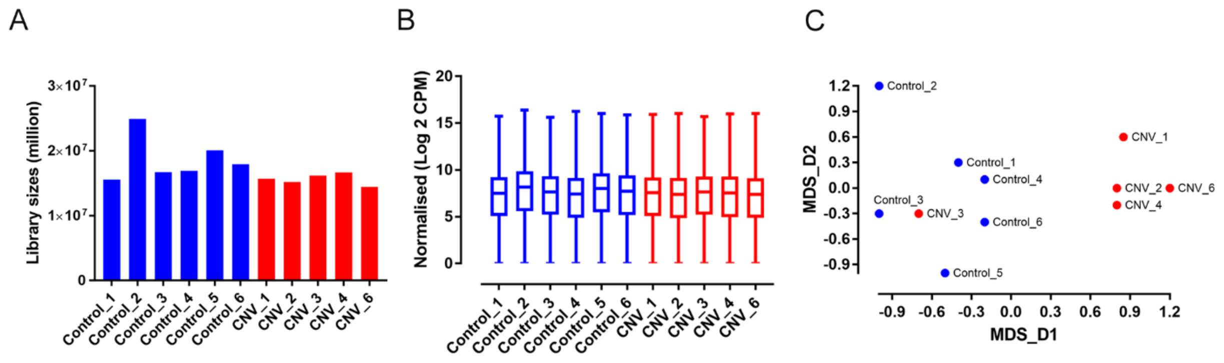

(A) Bar graph of library sizes that show similar sizes for control (blue) and CNV (red) samples. (B) Box and whiskers plot that show equivalent normalised gene expression (Log 2 CPM) between control (blue) and CNV (red) samples. (C) A MDS plot of the edgeR data of 500 genes per sample that shows two clusters for control (blue) and CNV (red) samples. CNV: choroidal neovascularisation, CPM: counts per million, MDS: multidimensional scaling

Table S6: mRNA expression of retina and RPE/choroid tissue (white box = CNV, grey box = CNV+OCX063)

Retina			RPE		
Gene	Fold Change	p-value	Gene	Fold Change	p-value
Edn1	1.7	0.037	Anpep	1.7	0.014
Csf3	-2.8	0.024	Akt1	-1.3	0.048
Fgfr3	-1.8	0.043	Bcl2	-2.4	0.029
Mdk	-1.4	0.019	Ccl3	2.2	0.024
Mmp19	-1.6	0.02	Col1a2	1.6	0.008
Sp1	-1.2	0.006	Cxcl1	1.9	0.006
Akt1	1.2	0.04	F2	-2	0.051
Bcl2	1.3	0.022	Flt1	-1.8	0.009
Cebpb	1.7	0.021	Il13ra2	1.7	0.019
Ilk	1.1	0.045	Itgb3	1.4	0.043
Itgb1	1.3	0.028	Jun	-1.8	0.032
Itgb5	1.4	0.039	Lox	2.8	0.014
Mmp14	1.5	0.011	Mmp13	2	0.014
Mmp2	1.2	0.033	Mmp8	4.1	0
Nfkb1	1	0.044	Plat	1.5	0.013
Plat	1.3	0.05	Smad4	-2.5	0.025
Smad3	1.2	0.047	Tgfb1	1.4	0.004
Smad4	1.3	0.025	Tgif1	-2.2	0.03
			Thbs1	1.6	0.042
			Timp1	3.4	0.014
			Epas1	-1.3	0.013
			Cebpb	1.3	0.032
			Ctgf	-1.3	0.062
			Ifng	2.3	0.048
			Igf1	-1.3	0.016
			Il10	1.7	0.04
			Itgav	-1.3	0.018
			Mdk	-1.5	0.026
			Mmp14	1.3	0.003
			Mmp3	1.4	0.002
			Thbs1	-1.3	0.031
			Timp1	-1.6	0
			Timp2	1.2	0.043

Table S7: Genes overlapped in expression between RNAseq and qPCR expression

qPCR			RNAseq				
**Gene**	**FC**	**P-value**	**Gene**	**logFC**	**FC**	**P-Value**	**FDR**
**Anpep**	1.7	1.36E-02	Anpep	1	2	1.21E-05	3.72E-04
**Ccl3**	2.2	2.36E-02	Ccl3	2.4	5.4	1.32E-07	8.14E-06
**Col1a2**	1.6	8.20E-03	Col1a2	1.7	3.3	1.19E-06	5.12E-05
**Il13ra2**	1.7	1.86E-02	Il13ra2	0.9	1.9	5.30E-03	4.62E-02
**Lox**	2.8	1.43E-02	Lox	1.9	3.7	2.08E-10	3.18E-08
**Mmp13**	2	1.38E-02	Mmp13	3.8	13.6	1.55E-07	9.29E-06
**Mmp8**	4.1	6.00E-05	Mmp8	2.1	4.4	2.76E-03	2.90E-02
**Tgfb1**	1.4	4.18E-03	Tgfb1	1.3	2.4	1.65E-10	2.58E-08
**Thbs1**	1.6	4.19E-02	Thbs1	0.8	1.8	4.40E-04	7.13E-03
**Timp1**	3.4	8.73E-04	Timp1	2.7	6.5	2.88E-19	2.64E-15

Figure S2: mRNA expression of RPE/choroid tissue that underwent laser induced photocoagulation

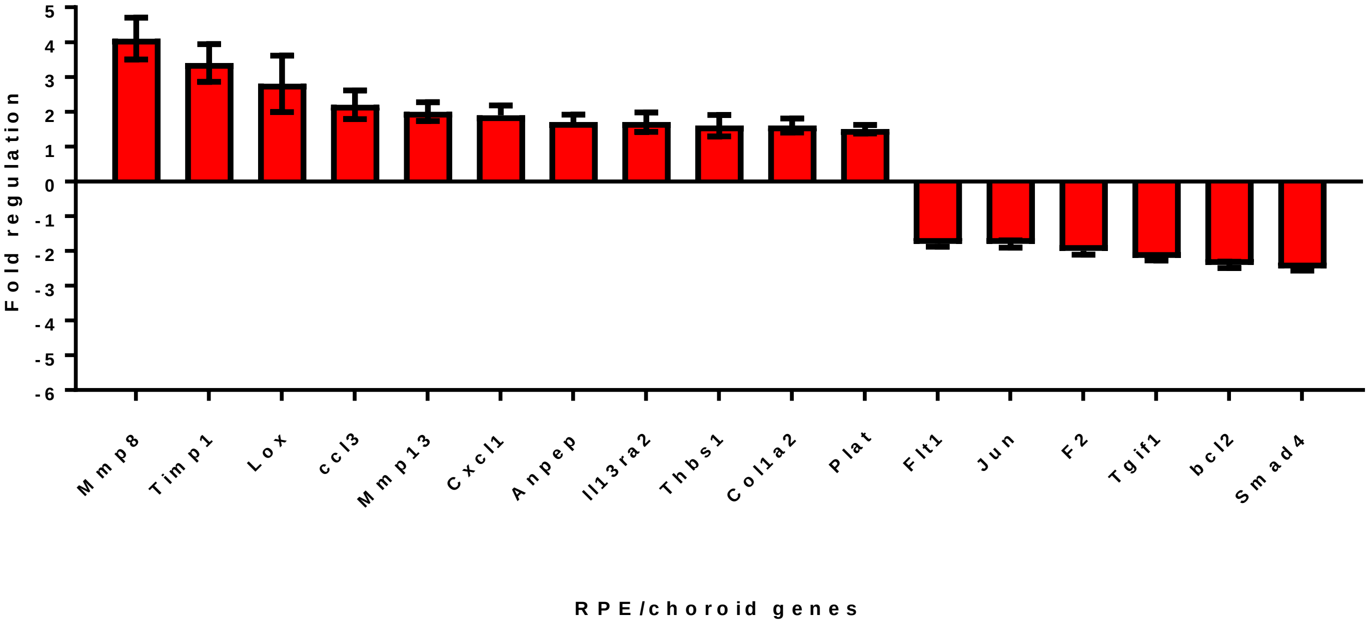

Seven days after laser induced photocoagulation RPE/choroid tissue separated, RNA was extracted and reverse transcribed. mRNA expression using Qiagen RT-profiler arrays shows that most gene expression (fold regulation, red bars) were upregulated compared to down regulated.
